# Intensity of mental health treatment of cancer-related psychopathology: the predictive role of Early Maladaptive Schemas

**DOI:** 10.1007/s00520-023-07764-w

**Published:** 2023-05-08

**Authors:** Irene H. de Vlaming, Melanie P. J. Schellekens, Marije L. van der Lee

**Affiliations:** 1 Department of Scientific Research, Helen Dowling Institute, Bilthoven, The Netherlands; 2 Department of Medical Psychology, Alrijne Hospital, Leiden, The Netherlands; 3 Department of Medical and Clinical Psychology, Center of Research on Psychology in Somatic Diseases, Tilburg University, Tilburg, The Netherlands

**Keywords:** Early maladaptive schema, Cancer, Psychological treatment, Mental disorders

## Abstract

**Purpose:**

With the limited availability of mental healthcare, it is of utmost importance to provide care that matches the needs of patients: short if possible, but also more intense when necessary. This study explored whether Early Maladaptive Schemas (EMSs) play a predictive role in the intensity of needed mental health treatment of cancer-related psychopathology.

**Methods:**

EMSs were assessed before mental health treatment in 256 patients who sought help at a specialized mental health care centre for those affected by cancer in the Netherlands. Data about treatment indication and intensity of mental health treatment were collected. Univariate and multivariate logistic regression analysis were used to assess the predictive value of the EMSs total score and specific domains on treatment indication and treatment intensity.

**Results:**

The presence of more severe EMSs predicted an indication for a more intense mental health treatment before start of the treatment, and actual more intense mental health treatment. The domain Impaired Autonomy and Performance appeared to be conceptually close to the domain Disconnection and Rejection, we left the latter out in our multivariate analysis and then found that Impaired Autonomy was the best predictor of intensity of mental health treatment.

**Conclusion:**

Our findings imply that assessing EMSs could help to identify patients who will receive more treatment time.

## Introduction

When confronted with cancer, patients face numerous stressors in all life domains, which can be very demanding. The majority of patients with cancer is remarkably resilient. Although they naturally experience some distress, these feelings do not persist and these patients do not develop psychological disorders [1, 2]. However, a substantial minority of patients with cancer develop persistent psychological problems that meet the criteria of mental disorders [3]. Anxiety disorders, mood disorders and posttraumatic stress disorders are more common after cancer than in the general population [2–6]. Increased rates of anxiety tend to persist for up to 10 years or more, whereas increased rates of depression are less long-lasting [4]. PTSD symptoms can also persist for many years or develop with delayed onset [7, 8].

Besides the fact that these persisting mental disorders can disrupt the lives of patients and their families, they also complicate the management of cancer [9]. Therefore, it is essential for patient management to identify patients at risk for psychopathology [10]. When patients at risk for mental problems are identified, they can be offered evidence-based treatments (e.g., [11–15]). Elevated distress in the context of cancer does not always imply a risk for psychopathology or need for psychosocial care [1, 16], because it is often a normal and transient reaction to stressful circumstances. In fact, low distress levels could in some cases be regarded as an abnormal reaction that warrants attention from professionals since it may indicate a shock reaction or denial. Therefore, screening for distress seems a rather blunt instrument to identify those needing psychological support, screening can be improved by identifying patients at risk [1]. One way to improve the identification of patients at risk would be to take the role of pre-existing vulnerability factors into account as part of the screening process.

In the general population unconditional core beliefs or schemas are known vulnerability factors for high levels of distress associated with persistent psychopathology [17, 18]. It is known that after a cancer diagnosis, secure attachment is predictive of a better working alliance with caregivers, greater perceived support, less general distress, greater confidence, and greater satisfaction compared to patients with an insecure attachment style [19, 20]. While insecure attachment style appeared to predict more difficulties handling diagnosis and treatment [21]. Inspired by attachment theory [22], the Schema Theory model emphasizes that all individuals are born with core emotional needs (i.e., secure attachment, autonomy, and realistic limits) [18]. When these core emotional needs are frustrated during childhood one can develop strongly held beliefs that are called Early Maladaptive Schemas (EMSs). EMSs contain unconditional themes regarding oneself, relationships, and the world (e.g., “I am incompetent”, “Others are unreliable”), that develop early in life and are elaborated throughout life into self-perpetuating cognitive structures [18]. In the Schema Theory model, EMSs are clustered in domains that have hypothesized associations with need-thwarting parental experiences in childhood. For example, when a parent is suffering from severe illness this can unintentionally cause limited predictable emotional attachment, leading to EMSs in the domain of rejection in the child. A schema-organization in four domains has been found empirically and conceptually consistent with the Schema Theory model [23]: Disconnection and Rejection, Impaired Autonomy and Performance, Excessive Responsibility and Standards, and Impaired Limits (Table 1). Fortunately, psychotherapy can change EMS severity and subsequently relief the associated symptomatic distress [24].

**Table 1:** Organization of the 16 EMSs in four domains

Domains Schema	A. Disconnection and rejection	B. Impaired autonomy and performance	C. Excessive responsibility and standards	D. Impaired limits
1	Emotional Deprivation	Dependence	Self-sacrifice	Entitlement
2	Social isolation/alienation	Failure	Unrelenting standards	Insufficient Self-control
3	Emotional inhibition	Sugjugation		
4	Defectiveness	Abandonment/instability		
5	Mistrust	Enmeshment		
6		Vulnerability to harm and illness		

The role of EMSs as vulnerability factors in developing persistent mental health problems in the context of cancer has been emphasized in a transdiagnostic model [25]. The challenges of cancer present an opportunity for (1) schema *disruption* when pre-existing beliefs are undermined after a cancer diagnosis (for example beliefs about excessive responsibility or grandiosity), or (2) schema *activation* when former adaptations to EMSs are reinforced by the impact of cancer (for example beliefs about defectiveness or vulnerability to harm). Both can lead to mental health problems with persistent distress [25–27]. More severe EMSs may also challenge the formation of an effective therapeutic relationship, since EMSs reflect self-perpetuating beliefs associated with frustrated emotional needs [18]. Consequently, more intense treatment is needed when patients suffer from severe EMS.

The objective of the present study is to examine whether EMSs are indeed associated with intensity of the mental health treatment in patients with cancer, reflecting more severe or more complex cancer-related psychopathology. We hypothesize (1) that more severe EMSs *predict an indication* for more intense mental health treatment; and (2) that patients with more severe EMSs *receive* more intense (i.e. more time consuming) mental health treatment. Based on our clinical expertise in psycho-oncology we expected a positive association between high scores on EMSs in the Disconnection and Rejection domain and the intensity of treatment, because challenges in dealing with cancer can lead to adjustment problems and interpersonal problems (including the therapeutic relationship) if basic trust is not established securely. Second, we expected a positive association between high scores on EMSs in the Impaired Autonomy and Performance domain and the intensity of psychological treatment because cancer and its consequences are very demanding. Feelings of being incapable or too vulnerable to deal with all these challenges can easily arise, which can also lead to higher needs of guidance in the therapeutic relationship. Third, we expected a positive association between high scores on EMSs in the Excessive Responsibility and Standards domain and the intensity of psychological treatment since uncertainty is inherent to cancer. This must be dealt with, while holding on to usual goal setting in daily life is often frustrated. EMSs in this domain may complicate coping with normal aspects of fear of cancer recurrence, leading to extended psychological treatment. We did not form any explicit hypotheses regarding the association between EMSs in the Impaired Limits Domain and intensity of psychological treatment.

## Methods

### Study design

We conducted a prospective cohort study at the Helen Dowling Institute (HDI), a mental healthcare institute for cancer patients and their loved ones, from December 2019 to April 2020.

### Participants and procedures

The study population consisted of adults suffering from different types of cancer, who were referred to the HDI for cancer-related mental health problems (they will be called clients from here). All clients were asked to fill in an additional questionnaire to assess EMSs (SQ-sf) [28] as part of the intake procedure. Data about treatment indication and intensity of mental health treatment were collected from electronic patient files. Mental health professionals were blinded for EMSs (SQ-sf) results.

### Measures

#### Client characteristics

Sociodemographic (i.e., sex, age, education level) and clinical characteristics (i.e. type and stage of cancer, current anti-cancer treatment) were obtained from clients’ files.

### Predictor

#### Early maladaptive schema

The 80-item short form of the Dutch version of the Young Schema Questionnaire (SQ-sf) [28] was used to measure EMSs. The SQ-SF is a self-report inventory profiling the intensity of 16 EMSs, that can be divided in 4 domains (see Table 1). Accordingly, respondents were required to rate each item on a six-point scale (from “completely untrue for me” to “describes me perfectly”). The questionnaire has been validated in a Dutch sample and shows good to excellent psychometric properties, making the instrument suitable for clinical practice [28].

### Outcome measures

#### Treatment indication

**Fig. 1:**
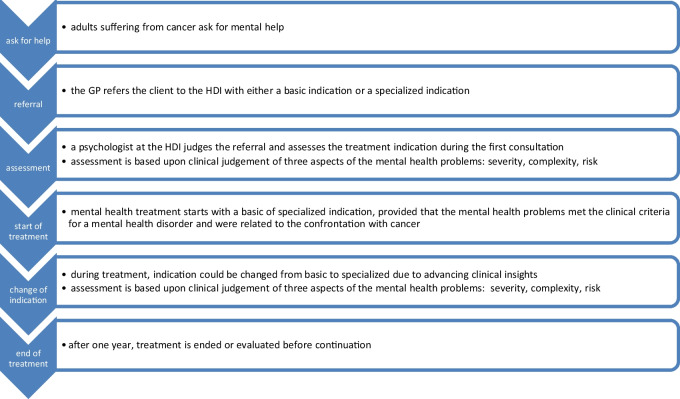
Prevailing model of mental healthcare used in this study.

In the Netherlands, clients are referred for mental health care by their general practitioner (GP). Following the prevailing model of health care at that time (see Fig. 1), GP’s needed to refer either for a basic treatment (so called Basic GGZ, roughly a treatment with a duration of < 800 min) or for a specialized treatment (so called Specialized GGZ, roughly a treatment with a duration of ≥ 800 min), respectively called ‘basic indication’ and ‘specialized indication’. At the HDI, the mean number of sessions after the intake in Basic GGZ was eight.

During the intake interview a clinical psychologist judged if the indication from the GP fitted with their expectation or whether it should be changed. This assessment is based upon clinical judgement of three aspects of the mental health problems: severity, complexity, and risk. If criteria for a mental health disorder (primary diagnosis) following DSM-5 [29] was not met, the client would be referred back to their GP for other forms of help. Provided that the mental health problems met the clinical criteria for a mental health disorder and were related to the confrontation with cancer, mental health treatment at the HDI was conducted.

Of clients who did not receive a primary diagnosis and time spend was less than 400 min, it was assumed that besides the intake interview no treatment took place. Therefore, these clients were excluded from the analysis. Some former basic indications could be converted to specialized indications during treatment, due to advancing clinical insights. In these cases, consultation took place among colleagues in a multidisciplinary meeting to rejudge the three aspects of psychopathology (severity, complexity, risk). The outcome was registered in the clients’ files.

### Intensity of mental health treatment

For treatment intensity, we used the sum of all minutes spent on the treatment as recorded in the clients’ files. This sum included time spent on treatment sessions, multidisciplinary meetings, psychiatric assessments, additional consultations and time spent on making notes of the sessions. Following the prevailing model of health care in the Netherlands at that time, we divided treatment intensity into two groups: (1) < 800 min (Basic GGZ) and (2) ≥ 800 min (Specialized GGZ), respectively called ‘low intensity’ and ‘high intensity’ from here. It is important to note that therapists are stimulated to end treatment as soon as treatment goals are reached. There is a waiting list with patients who are urgently waiting for care and therapists are aware that if a client is resilient enough to end therapy, they will be able to help another client. Furthermore, the prevailing model of health care paid fixed prices for treatment and not per session, also stimulating time efficiency.

### Data analyses

Statistical analyses were performed using SPSS version 25. Univariate logistic regression analysis was used to assess the predictive value of the EMSs total score and specific domains on treatment indication (basic versus specialized) and treatment intensity (low versus high). Furthermore, within the group of short treatment indications, we assessed the predictive value of the total score of EMSs and specific domains on whether the short indication was maintained or changed to intensive indication during treatment. When multiple domains proved to be a significant predictor of treatment indication or treatment intensity, they were added simultaneously in a multivariate logistic regression model to assess the relative predictive value of the domains. In addition, we explored whether the domains were predictive above and beyond sociodemographic and clinical characteristics: age, gender, education level and prognosis.

The analysis plan was pre-registered at OSF: https://osf.io/hdwuj/.

## Results

### Study sample

Between December 2019 and June 2020, clients referred to the HDI for cancer-related mental health problems were invited to fill out the SQ-sf [28] at admission. Of these 510 clients, 417 clients (81.8%) agreed to participate. Clients were excluded, because they were close others of cancer patients (*n* =96, 23%), there was no primary diagnosis (*n* = 57, 14%), or we were unable to follow them up for 12 months of mental health treatment (*n* =1, 0.2%). Data of the resulting 256 patients were used for analysis (see Table 2).

**Table 2:** Sociodemographic and clinical characteristics of 256 clients

	*N* (%)
Sociodemographic characteristics	
Gender, women	177 (69.1)
Age, M (SD)	49.58 (12.4)
Education level	
Low	22 (8.6)
Middle	96 (37.5)
High	138 (53.9)
Living situation	
By oneself	41 (16.0)
With children	14 (5.5)
With partner	95 (37.1)
With partner and children	96 (37.5)
Differently	10 (3.9)
Job status*	
Paid job	124 (48.1)
Called in sick	113 (44.1)
Incapacitated	42 (16.4)
Retirement	33 (12.9)
Takes care of household	63 (24.6)
Student	9 (3.5)
Volunteering work	22 (8.6)
Clinical characteristics	
Type of cancer*	
Breast	105 (41.0)
Digestive system	32 (12.5)
Lung	16 (6.3)
Hematological	30 (11.7)
Genital organs	34 (13.3)
Other	
Time since diagnosis	
0–6 months	77 (30.1)
7–12 months	57 (22.3)
1–2 years	41 (16.0)
2–5 years	52 (20.3)
> 5 years	29 (11.3)
Treatment*	
Surgery	179 (69.9)
Chemotheray	164 (64.1)
Radiotherapy	117 (45.7)
Hormone treatment	55 (21.5)
Immunotherapy	43 (16.8)
Other treatment	47 (18.3)
Prognosis	
Cured	81 (31.6)
Good chance of survival	101 (39.4)
Little/no chance of survival	74 (28.9)

Table 3 shows the total, domain, and separate EMS scores. In clinical practice, a cut-off of 2.5 is considered optimal for detecting the presence of clinically significant EMSs, except for Defectiveness (2.0), Subjugation and Unrelenting standards (both 3.0) [30, 31].

**Table 3:** Total, domain, and EMS scores of 256 clients

	M (SD)
Total EMS	2.21 (0.76)
Domain disconnection and rejection	2.00 (0.84)
Emotional deprivation	1.96 (1.00)
Social isolation	2.17 (1.09)
Emotional inhibition	2.12 (0.93)
Defectiveness	1.65 (0.87)
Mistrust	2.09 (1.01)
Impaired autonomy and performance	2.15 (0.80)
Dependence	2.30 (0.98)
Failure	2.04 (1.09)
Subjugation	2.42 (1.08)
Abandonment	2.06 (1.07)
Enmeshment	1.74 (0.92)
Vulnerability to harm/illness	2.36 (1.02)
Excessive responsibility and standards	2.84 (1.05)
Self-sacrifice	3.00 (1.18)
Unrelenting standards	2.69 (1.12)
Impaired limits	2.41 (0.88)
Entitlement	2.52 (0.99)
Insufficient self-control	2.31 (0.92)
Not part of a domain	
Social Undesirability	1.90 (0.86)

### Multicollinearity in schema domains

Since the correlation among domains was large (*r* between .59 and .81, *p* values < .001) we performed ordinary least squares regression with the four domains as predictors to obtain the indices of the impact of multicollinearity on the precision of estimation. For the domains Disconnection and Rejection and Impaired Autonomy and Performance we found a heightened variance inflation factor above the cut-off of 2.5 for logistic regression (3.35 and 3.76, respectively) [30–32]. In addition, a condition index of > 10 indicates the presence of multicollinearity (i.e., 14.42). It showed high variance proportions of both domains’ regression coefficients (i.e., .57 for Disconnection and Rejection and .92 for Impaired Autonomy and Performance). These findings indicate that multicollinearity threatened the stability of the estimates. Therefore, when both domains showed significant predictive value in univariate analysis, only Impaired Autonomy and Performance was carried forward to the multivariate model. We choose this domain because it includes the EMS Vulnerability to Harm and Illness. Elevation of this EMS is considered highly relevant for our clients. Since EMSs are more than simply a reflection of symptoms [33], being confronted with cancer in real life must be extra stressful for those with this EMS as an underlying vulnerability factor.

### EMSs predicting treatment indication

Of the 256 clients, 142 clients (55.5%) received a basic indication, and 114 clients (44.5%) received a specialized indication, which is representative for referrals to the HDI in previous years. Clients with a higher total score on the SQ-sf were more likely to receive a specialized than basic indication (OR = 2.17; 95% CI = 1.52–3.10; *p* < 0.001). Univariate analyses (see Table 4A) show that patients with higher scores on either domain were significantly more likely to have received a specialized rather than a basic indication (all *p’s* < 0.01), in accordance with our hypotheses. Adding all contributing univariate factors in a multivariate logistic regression model (with the exception of Disconnection and Rejection due to multicollinearity) showed that none of the domains remained independently associated with treatment indication. Adding gender, age, education level and prognosis to the multivariate model with domains showed similar results.

**Table 4:** Predictive value of domains related to (A) treatment indication (basic versus specialized), (B) changes in treatment indication (remained basic versus changed to specialized) and (C) treatment intensity (low versus high intensity)

	Basic (*n* = 142)	Specialized (*n* = 114)	Univariate analysis		Multivariate analysis	
A	*M* (SD)	*M* (SD)	OR (95% CI)	*P*	OR (95% CI)	*P*
Disconnection and Rejection	1.79 (0.73)	2.25 (0.89)	2.00 (1.45, 2.77)	< .001	*	*
Impaired Autonomy and Performance	1.97 (0.70)	2.39 (0.85)	2.00 (1.43, 2.81)	< .001	1.65 (0.98, 2.79)	.059
Excessive responsibility and standards	2.62 (0.96)	3.12 (1.10)	1.60 (1.25, 2.06)	< .001	1.24 (0.87, 1.76)	.229
Impaired limits	2.26 (0.82)	2.59 (0.91)	1.56 (1.16, 2.09)	.003	0.99 (0.65, 1.49)	.951
	Remain basic (*n* = 89)	Changed to specialized (*n* = 53)				
B	*M* (SD)	*M* (SD)				
Disconnection and rejection	1.72 (0.67)	1.93 (0.82)	1.47 (0.93, 2.35)	.103		
Impaired autonomy and performance	1.82 (0.59)	2.21 (0.81)	2.21 (1.31, 3.73)	.003		
Excessive responsibility and standards	2.51 (0.90)	2.81 (1.04)	1.39 (0.97, 2.00)	.072		
Impaired limits	2.19 (0.75)	2.39 (0.93)	1.33 (0.88, 2.01)	.175		
	Low intensity (*n* = 88)	High intensity (*n* = 168)				
C	*M* (SD)	M (SD)				
Disconnection and rejection	1.72 (0.66)	2.14 (0.88)	2.02 (1.39, 2.92)	< .001	*	*
Impaired autonomy and performance	1.86 (0.60)	2.31 (0.85)	2.27 (1.53, 3.37)	< .001	2.09 (1.14, 3.81)	.016
Excessive responsibility and standards	2.52 (0.90)	3.01 (1.09)	1.62 (1.24, 2.12)	< .001	1.16 (0.79, 1.70)	.458
Impaired limits	2.20 (0.77)	2.52 (0.91)	1.60 (1.15, 2.21)	.005	0.92 (0.59, 1.45)	.731

*Domain has been left out multivariate analysis due to multicollinearity

### EMSs predicting changes in treatment indication during treatment

Of the 142 basic indications 89 (62.7%) indications remained unchanged while 53 (37.3%) indications were converted to specialized indications during treatment. Among clients with a basic indication, those with a higher total SQ-sf score were more likely to have their indication changed to specialized than to maintain the basic indication (OR = 1.83; CI = 1.10–3.04; *p* = 0.020). Univariate analyses (see Table 4B) showed that patients with higher scores on Impaired Autonomy and Performance were significantly more likely to receive a specialized than basic indication (all *p* < 0.01). None of the other domains were significant predictors of changes in treatment indication. Adding gender, age, education level and prognosis to the multivariate model with domains showed similar results.

### EMSs predicting intensity of mental health treatment

Of 256 clients, 88 (34.4%) clients received treatment with a low intensity while 168 clients received a treatment with a high intensity. Also, in line with our hypotheses, clients with higher total scores on the SQ-sf were significantly more likely to receive a treatment with a high intensity (OR = 2.30; CI = 1.54–3.42; *p* < 0.001). Univariate analyses (see Table 4C) showed that patients with higher scores on either domain were significantly more likely to receive a treatment with a high intensity than a low intensity (all *p’s* < 0.01). Adding all contributing univariate factors in a multivariate logistic regression model (with the exception of Disconnection and Rejection due to multicollinearity) revealed that Impaired Autonomy and Performance remained independently associated with treatment intensity (*p* = 0.016). Adding gender, age, education level and prognosis to the multivariate model with domains showed similar results.

## Discussion

The present study found that the presence of more severe EMSs predicted (1) an indication for a more intense mental health treatment before start of the treatment, and (2) actual more intense mental health treatment, meaning more minutes spent on treatment. These more intense treatments started either with an indication for specialized treatment, or were, in almost half of the cases, converted from a basic to a specialized indication during mental health treatment.

All schema EMS domains (Disconnection and Rejection, Impaired Autonomy and Performance, Excessive Responsibility and Standards, and Impaired Limits) were independently relevant predictors of treatment indication (basic versus specialized) and treatment intensity (low or high intensity). These findings supported most of our predictions, except that a priori hypothesis about the domain Impaired Limits had not been specified due to missing clinical expertise about its relation to cancer-related psychopathology. When we explored the predictive role of all domains simultaneously, the domain Impaired Autonomy and Performance seemed the strongest predictor of intensity of treatment. Note that, due to multicollinearity, we removed the domain Disconnection and Rejection from the model.

The domain Impaired Autonomy and Performance reflects the level of confidence people have in their capacity to deal with difficulties. High scores in this domain implicate little self-confidence and higher needs of guidance when confronted with challenges. A cancer diagnosis and its treatment can easily trigger these feelings and needs. Usually, it takes time to adjust to a cancer diagnosis and lengthy medical treatment, so one’s sense of competence is tested for a longer period of time. Feelings of incapability that arise from EMSs in this domain can easily lead to more intense mental health treatment. Moreover, accomplishing and letting go of psychological treatment that offers support in this context can be extra challenging for those who need more guidance and support, also leading to extended mental health treatment. This finding supports previous research showing that lower self-efficacy for coping with a cancer diagnosis is related to heightened distress and poorer adjustment to cancer [34].

## Clinical implications

With the limited availability of mental healthcare, it is of utmost importance to provide care that matches the needs of patients: as short as possible, but also more intense when necessary. EMSs and EMS domains could offer valuable input in helping (1) to identify what patients need more intensive mental health treatment and (2) to determine what treatment would be most beneficial for the patient, addressing high EMSs and stimulating an effective therapeutic relationship. The content of the specific EMSs gives information about the needs of the patient and subsequently the sort of treatment that fits those needs best. Our findings might also be applicable outside of the mental health setting to the patients in the hospital, where EMSs might guide the medical team in how to support the patient. When this is done as part of the screening process early on in the disease trajectory this might help the medical team to know how to best support the patient during this often harsh period of medical treatment.

Our results imply that therapists should pay extra attention to clients scoring higher on EMSs, because they probably have higher needs of guidance in dealing with the challenges a cancer diagnosis poses. Interestingly, the interaction between therapist and client may have contributed to the present findings of EMS domains predictive role in more intensive treatment. Therapists might have offered more and longer lasting support in response to somewhat clinging behaviour of clients that originates from EMSs in the Impaired Autonomy and Performance domain. Unintentionally, therapists may have confirmed aspects of little self-efficacy, leading to more intense mental health treatment. When EMSs in this domain are elevated, supporting patients in increasing their self-efficacy may help overcome, for example, the client becoming too dependent on the therapist.

Schema therapy might serve as a source for possible interventions to target activated EMSs in the context of cancer [35]. A clear focus on the EMSs can contribute to effectively reducing schema activation or disruption.

## Study limitations

This is the first study that explored the role of EMSs in the context of mental health treatment among patients suffering from cancer. Some limitations need to be considered. The domain Impaired Autonomy and Performance appeared to be conceptually close to the domain Disconnection and Rejection. Consequently, it is not sure if one domain is a better predictor of intensity of mental health treatment than the other. These domains together conceptually reflect a broad spectrum of vulnerabilities in a sense of self-confidence, self-acceptance and identity.

The study sample was a heterogeneous group of adults with different types and stages of cancer who were referred for psychological care and contacted the HDI themselves. This limits the generalizability of the findings to cancer patients who suffer from psychopathology but are less motivated to seek help. As this study was embedded in routine clinical practice, these findings do generalize to patients seeking help.

Another limitation is that we only obtained fairly general information about treatment intensity, because treatment intensity was divided in two groups of minutes spent on treatment. Since this division followed the prevailing model of mental healthcare in the Netherlands at that time, it is conceivable that treatment intensity was influenced by these rules.

## Implications for future research

Further research is needed to explore whether mental health treatment leads to reducing EMSs in the domains that predicted mental health treatment intensity, improving the more persistent symptoms of distress in the context of cancer. More insight in what EMS contributes specifically to the predictive value of the schema domains can offer valuable input for tailoring psychological treatment. Furthermore, this study revealed that vulnerabilities in a sense of autonomy and performance predicted a more intense treatment but interacting therapist factors remained unclear. It would be interesting to study this interaction and to what extent the therapeutic relationship contributes to the intensity of mental health treatment.
